# The Burden of Obesity and Type 1 Diabetes: Distinct and Synergistic Effects to Worsen Perceived Health and Psychosocial Distress

**DOI:** 10.1111/dom.70629

**Published:** 2026-03-09

**Authors:** Renata Risi, Domiziana Pauletto, Valeria Zanghi Buffi, Valentina Berna, Andrea Carafa, Federica Barbaro, Francesco De Vita, Luca D'Onofrio, Rocco Amendolara, Angelo Lauria Pantano, Alessandra Di Flaviani, Simona Zampetti, Dario Tuccinardi, Raffaella Buzzetti, Ernesto Maddaloni, the AD_OWOB study group

**Affiliations:** ^1^ Department of Experimental Medicine Sapienza University of Rome Rome Italy; ^2^ Department of Endocrinology and Diabetes University Campus Bio‐Medico of Rome Rome Italy; ^3^ Diabetology Unit Policlinico Umberto I Rome Italy; ^4^ Azienda Ospedaliera San Camillo Forlanini Rome Italy; ^5^ Ordine dei Cavalieri di Malta Rome Italy; ^6^ Fondazione Policlinico Universitario Campus Bio‐Medico Rome Italy

**Keywords:** obesity care, observational study, patient reported outcomes, perceptions, type 1 diabetes

## Abstract

**Background:**

Type 1 diabetes (T1D) and overweight/obesity (OW) are chronic conditions impairing health‐related quality of life (HRQoL). While diabetes primarily affects psychosocial domains, OW impacts physical functioning and social stigma. Despite the increasing prevalence of OW among individuals with T1D, their combined effect on HRQoL has not been investigated.

**Methods:**

In this cross‐sectional, multicentre study we evaluated HRQoL in 43 people with both T1D and OW (T1DOW), 40 people with T1D normal weight (T1DNW), 58 normal weight people without diabetes (NADNW) and 41 people with OW without diabetes (NADOW). All participants completed generic HRQoL questionnaires (WHO‐5, SF‐36). People with T1D additionally completed diabetes‐specific tools (DDS, DES‐SF and T1‐DDAS). Adjusted multivariate analyses were used to evaluate the independent and interactive effects of T1D and OW on HRQoL.

**Results:**

T1D was associated with reduced well‐being (WHO‐5, *p* = 0.003), impaired general health perception (SF‐36, *p* < 0.001) and lower social functioning (SF‐36, *p* = 0.026). OW significantly worsened physical functioning (*p* < 0.001) and role limitations due to physical health (*p* = 0.036). Bodily pain was synergistically affected by both T1D (*p* = 0.043) and OW (*p* = 0.006), with people affected by both T1D and OW showing the worst score. Overall, T1DOW was associated with greater detrimental effects on WHO‐5 and SF‐36 outcomes compared to OW and T1D alone. Among people with T1D, OW selectively increased interpersonal distress (DDS, *p* = 0.020), while empowerment and T1D‐specific distress were unaffected.

**Conclusions:**

T1D and OW exert distinct, partially overlapping effects on HRQoL. Their coexistence worsens body pain and interpersonal distress and emphasises the need for integrated strategies in managing ‘double diabetes’.

## 1 Introduction

Evidence largely supports that type 1 diabetes (T1D) exerts a detrimental impact on health‐related quality of life (HRQoL). The DAWN study first documented that T1D leads to a significant deterioration across all domains of HRQoL, with only 19.4% of people with T1D fully adhering to therapeutic recommendations and 85.2% of newly diagnosed patients reporting intense negative emotions [1]. The subsequent DAWN2 study, conducted on 8596 adults with diabetes across 17 countries, confirmed and expanded these findings, showing that 13.8% of patients exhibited signs of probable depression, 44.6% reported significant diabetes‐related distress, diabetes affecting all aspects of life investigated, ranging from 20.5% for family relationships to 62.2% for physical health [2]. Specific studies have also highlighted notable gender differences, with women reporting significantly lower HRQoL (75 vs. 80 on a 0–100 scale, *p* < 0.05) and a higher prevalence of depression (31.7% vs. 14.9%) compared with men [3]. In parallel, scientific literature has consistently documented obesity as a chronic condition exerting a significant and multidimensional impact on HRQoL, comparable to that observed in other severe chronic diseases. A comprehensive meta‐analysis of 47 studies showed that adults with obesity reported significantly lower HRQoL scores than normal‐weight individuals across all domains, with a dose‐dependent relationship between obesity severity and HRQoL impairment [4]. This manifests through reduced physical functioning, associated with mood and anxiety disorders [5] and substantial social barriers driven by weight stigma [6]. The internalisation of such stigma further amplifies the negative impact through processes of self‐blame and shame [7]. Despite the increasing prevalence of obesity among individuals with T1D [8, 9], the synergistic impact of both conditions on QoL has been little investigated. Recent evidence has evaluated how obesity associated with T1D leads to impairments in quality of life across specific WHO‐5 and SF‐36 domains [10]. We hypothesise that both T1D and overweight/obesity are independently associated with reduced HRQoL, and that their coexistence may exert a synergistic effect on it. Therefore, in this study we primarily aimed to explore patient reported outcome measures (PROMs) about HRQoL in people with T1D and comorbid overweight or obesity, compared to people with T1D without overweight nor obesity. Secondarily, we also aimed to assess the synergistic effects of T1D and overweight/obesity on HRQoL by comparing results obtained in people with T1D to people without diabetes with and without a condition of overweight or obesity.

## 2 Methods

### 2.1 Study Design and Population

This cross‐sectional study was conducted at the Azienda Ospedaliero‐Universitaria Policlinico Umberto I, Sapienza University of Rome and at Campus Bio‐Medico University Hospital of Rome, from February 2024 to March 2025. Two hundred eighty Caucasian people aged 18–75 years, 70 for each of the following four study groups, were screened: (1) patients with T1D and normal weight (T1DNW), (2) patients with T1D and overweight/obesity (T1DOW), (3) people with normal weight and without diabetes (NADNW) and (4) people with overweight/obesity without diabetes (NADOW). T1D was defined according to American Diabetes Association criteria [11]. Overweight/obesity was defined in the presence of a BMI ≥ 25 kg/m^2^.

Exclusion criteria were: chronic glucocorticoid therapy; pregnancy; liver cirrhosis; previous bariatric surgery; cancer diagnosis in the past 5 years; inability to complete questionnaires due to language or cognitive barriers.

### 2.2 Patient Reported Outcome Measures (PROMs)

The World Health Organization‐five well‐being index (WHO‐5) and the Short Form 36 Health Survey (SF‐36) were administered to all study participants to evaluate mental well‐being and general health perception, respectively [12, 13].

Three diabetes‐specific questionnaires (Diabetes Empowerment Scale—Short Form, DES‐SF; Type 1 Diabetes Distress Assessment System, T1‐DDAS; Diabetes Distress Scale, DDS) were administered only to people affected by T1D to capture specific aspects of the experience of living with diabetes [14, 15, 16]. Questionnaires were administered in person or sent by email. To maximise response rates, participants were contacted by phone up to two times in 3 months as reminders. Only patients having completed all questionnaires were included in the analysis.

### 2.3 Data Collection

Demographic data (age, sex, smoking status categorised as never, current, or former smoker), physical activity level (> 3.5 or ≤ 3.5 h/day), presence of hypertension and dyslipidaemia and current drug therapy were self‐reported by study participants. Weight and height were measured at enrolment while participants were wearing light clothes. Body mass index was calculated by dividing weight in kilograms by squared height in metres.

Data regarding diabetes history, co‐morbidities, biochemistry (HbA1c, total cholesterol, triglycerides, HDL cholesterol, LDL cholesterol, serum creatinine, eGFR and urine albumin) were collected only for people with T1D from electronic clinical records in the previous 6 months. Estimated glomerular filtrate rate (eGFR) was calculated with the Chronic Kidney Disease Epidemiology Collaboration (CKD‐EPI) formula. Microalbuminuria was defined for urine albumin levels > 30 mg/L. Diabetic retinopathy was defined in the presence of mild non‐proliferative diabetic retinopathy or more severe stages. Specific data on therapeutic management were collected, including disease duration (months), insulin therapy regimen (basal only/multiple daily injections/continuous subcutaneous insulin infusion [CSII]), daily insulin dose (total and per kg body weight, divided into basal, rapid‐acting and total units) and concomitant use of oral antidiabetic agents.

### 2.4 Statistical Analysis and Sample Size Calculation

Continuous variables are presented as median [25th–75th percentile] and categorical variables as number and percentage. The Shapiro–Wilk test was used to assess the distribution of continuous variables. The Kruskal–Wallis test was used to evaluate differences in continuous variables among groups. Categorical variables were analysed with the Chi‐square test or Fisher's exact test, as appropriate. To assess the impact of T1D and OW on general HRQoL questionnaires (WHO‐5, SF‐36), multivariate regression analyses were performed, accounting for sex and age (model 1) and sex, age, diagnosis of hypertension and dyslipidaemia (model 2) as confounders. To assess the impact of OW on diabetes‐related PROMs (DES‐SF, T1‐DDAS and DDS), multivariate regression analyses were performed with sex, age disease duration and type of insulin therapy (multiple daily injections [MDI] or CSII) as potential confounders. Briefly, the results of the questionnaires were used as dependent variables, diabetes and overweight/obesity status as main exposures and confounders were tested in the multivariate model and retained at a conservative retention *p*‐value < 0.1. For general psychological health questionnaires (WHO‐5 and SF‐36), an interaction analysis was also conducted to evaluate whether T1D and overweight/obesity interacted on the outcome. Non‐parametric variables were log‐transformed before entering the model.

To evaluate the impact of autoimmune diabetes and overweight/obesity on WHO‐5 scores in a 2 × 2 factorial design (four groups: T1DNW, T1DOW, NADNW and NADOW), assuming a 5‐point difference in WHO‐5, SD = 9, *α* = 0.05 and 80% power, approximately 35 participants per group were required [17, 18]. To guarantee the enrolment of the required number of participants completing all questionnaires, we anticipated that at least *n* * 2 (70) people for each study group had to be screened for participation in the study.

### 2.5 Ethics

The study was performed in accordance with the Declaration of Helsinki, and the study procedures were approved by the ethics committee ‘Lazio Area 1’ [Prot 0166/24]. All participants signed written informed consent.

## 3 Results

### 3.1 Population Characteristics

Of the 280 people screened for participation, 18 were excluded according to the inclusion/exclusion criteria, and 80 did not complete the questionnaires (Figure S1). The remaining 182 participants were distributed across the study groups as follows: 43 people affected by both T1D and OW (T1DOW), 40 normal weight people with T1D (T1DNW), 58 normal weight people without diabetes (NADNW) and 41 people with OW without diabetes (NADOW). Population features stratified by groups are summarised in Table 1.

**TABLE 1:** Basal characteristics of study population stratified by groups (NADNW, NADOW, T1DNW and T1DOW).

	NAD		T1D		*p*			
	Normal weight (*n* = 58)	Overweight (*n* = 41)	Normal weight (*n* = 40)	Overweight (*n* = 43)	NADNW vs. T1DNW	NADOW vs. T1DOW	T1DNW vs. T1DOW	NADNW vs. NADOW
Age, years	28 (25–53)	42 (35–47)	45 (32–56)	48 (36.5–55.25)	**0.0091**	0.056	0.95	**0.006**
Sex, F %	31 (64.6)	32 (62.7)	22 (55)	22 (51.2)	0.36	0.71	0.73	0.67
BMI, kg/m^2^	22.1 (20.5–23.8)	28.2 (24.9–30.6)	21.56 (20.52–23.8)	29.7 (26.71–32.45)	0.35	**0.004**	**< 0.0001**	**0.026**
CSII %	NA	NA	22 (55)	30 (69.8)	NA	NA	0.16	NA
Total insulin daily dose/kg	NA	NA	0.43 (0.38–0.52)	0.51 (0.45–0.55)	NA	0.36	NA	NA
Physical activities, *n* %	21 (43.75)	7 (13.72)	22 (55)	12 (27.9)	0.35	**0.088**	0.82	**0.0071**
Smokers, *n* %	6 (12.5)	13 (25.49)	15 (37.5)	7 (16.28)	**0.0061**	0.27	**0.03**	**0.09**
Hypertension, *n* %	1 (2.08)	6 (11.76)	12 (30)	17 (39.5)	**0.001**	**0.001**	0.52	**0.043**
Dyslipidaemia, *n* %	2 (4.17)	4 (7.84)	16 (40)	21 (48.8)	**< 0.0001**	**< 0.0001**	0.45	0.52
Rethynopathy, *n* %	NA	NA	6 (15)	6 (14)	NA	NA	0.99	NA
Albuminuria, *n* %	NA	NA	1 (2.5)	4 (9.3)	NA	NA	0.17	NA
Neuropathy, *n* %	NA	NA	2 (5)	3 (6.9)	NA	NA	0.32	NA
CV history, *n* %	0 (0)	1 (1.96)	0 (0)	3 (6.9)	NA	0.64	NA	NA
HbA1c, %	NA	NA	7.3 (6.7–8.32)	7.5 (7.09–8.55)	NA	NA	0.76	NA
Glycaemia, mg/dL	NA	NA	134 (96.5–166.3)	129 (101.5–180.5)	NA	NA	0.79	NA
Tot‐C, mg/dL	NA	NA	170.5 (151.5–186.8)	160.5 (136.5–173.8)	NA	NA	0.36	NA
HDL‐C, mg/dL	NA	NA	65.5 (51.25–77.25)	58 (44.68–68.25)	NA	NA	**0.03**	NA
LDL‐C, mg/dL	NA	NA	83.2 (70.85–110.6)	81.2 (73–103.4)	NA	NA	0.81	NA
Triglycerides, mg/dL	NA	NA	66 (48–90.75)	69 (54.5–92.5)	NA	NA	0.88	NA
eGFR, mL/min/1.73 m^2^	NA	NA	94.74 (79.42–110)	93.48 (84.5–105.8)	NA	NA	0.61	NA

*Note*: Bold values indicate stastistically significance (*p* value < 0.05).

Abbreviations: BMI, body mass index; CSII, continuous subcutaneous insulin infusion; CV history, cardiovascular history; eGFR, estimated glomerular filtration rate; HbA1c, glycated haemoglobin; HDL‐C, HDL cholesterol; LDL‐C, LDL cholesterol; NAD, non‐diabetes; NW, normal weight; OW, overweight/obese; T1D, type 1 diabetes; Tot‐C, total cholesterol.

Briefly, participants in the NADNW group were significantly younger than those in the T1DNW and NADOW groups (NADNW: age 28 [25–53] years; NADOW: age 42 [35–47] years; T1DNW: age 42 [35–47] years; T1DOW: age 48 [36.5–55.25] years; *p* = 0.0091 for NADNW vs. T1DNW; *p* = 0.056 for NADOW vs. T1DOW; *p* = 0.95 for T1DNW vs. T1DOW; *p* = 0.006 for NADNW vs. NADOW). Participants with T1DNW and NADNW showed similar BMI, while those with T1DOW had slightly higher BMI than those with NADOW (T1DOW: BMI = 29.7 (26.71–32.45) kg/m^2^ vs. NADOW: BMI = 28.2 (24.9–30.6) kg/m^2^, *p* value = 0.004). Sex distribution was homogeneous across the groups. People with T1DOW had lower HDL cholesterol levels than the NW counterpart [T1DNW: HDL Cholesterol = 65.5 (51.3–77.3) mg/dL vs. T1DOW: HDL Cholesterol = 58.0 (44.7–68.3) mg/dL, *p* value = 0.030], while smoking habit was more frequent in the latter compared to the first.

### 3.2 Impact of Autoimmune Diabetes and Overweight/Obesity on Quality of Life

**FIGURE 1:**
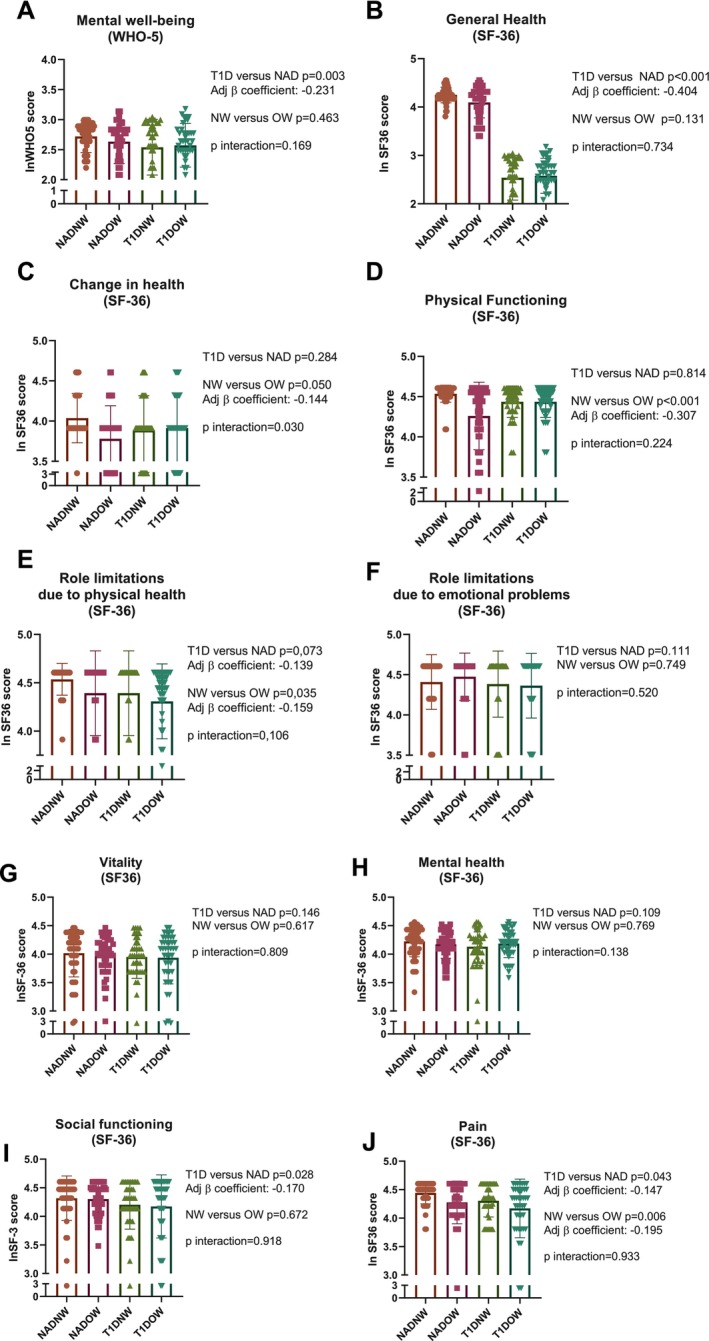
(A–J) Comparison of psychological well‐being and quality of life among groups. Measures include: WHO‐5 emotional well‐being; SF‐36 general health, perceived health change, physical functioning, role limitations due to physical health or emotional problems, vitality, mental health, social functioning and bodily pain. Analyses assessed the effects of diabetes (T1D vs. NAD), weight status (NW vs. OW) and their interaction. *β* coefficients are adjusted for sex, age and disease duration; *p*‐values are reported for each comparison. NAD, non‐diabetic; NADNW, non‐diabetic normal weight; NADOW, non‐diabetic overweight/obese; NW, normal weight; OW, overweight/obesity; SF‐36, Short Form Health Survey 36; T1D, autoimmune diabetes; T1DNW, T1D normal weight; T1DOW, T1D overweight/obese; WHO‐5, World Health Organization‐Five Well‐Being Index.

Figure 1 shows the comparison of the four groups across all HRQoL domains assessed by WHO‐5 and SF‐36.

#### 3.2.1 Mental Well‐Being (WHO‐5)

WHO‐5 scores (Figure 1A) were significantly lower among people with T1D compared to healthy controls after adjustment by age and sex (model 1, *p*‐value = 0.003; adjusted *β* = −0.231). In contrast, weight status did not significantly affect emotional well‐being (model 1, *p*‐value = 0.463), and no significant interaction between T1D and OW was detected (model 1, *p*‐value = 0.169).

After adjustment for the additional covariates included in model 2 (hypertension and dyslipidaemia), the association between T1D and lower WHO‐5 scores disappeared significantly (adjusted *β* = 0.082, adjusted *p*‐value = 0.742). Twelve out of 83 people with T1D (14.5%) scored below 50 on WHO‐5, a threshold suggestive of likely depression requiring clinical evaluation, compared with only 3 out of 99 healthy controls (3.0%) (*p*‐value = 0.0052).

#### 3.2.2 36‐Item Short Form Health Survey (SF36)

General health perception (Figure 1B) was significantly impaired in people with T1D compared to controls (model 1, *p* < 0.001; adjusted *β* = −0.404) in both people with and without OW (model 1, *p*‐value for interaction 0.734). This result was preserved also after adjustment for hypertension and dyslipidaemia (model 2, adjusted *p*‐value = 0.002, adjusted *β* = −0.514). Although lower values were observed in people with OW compared to people with normal weight, this difference was not statistically significant (*p* = 0.131).

For the SF‐36 item assessing perceived health change in the past year (Figure 1C), no significant differences emerged between T1D and controls (*p* = 0.284). However, OW was significantly associated with worse health change after adjustment by sex and age (model 1, *p* = 0.030; adjusted *β* = −0.144), although the significance was lost after additional adjustment for hypertension and dyslipidaemia (model 2, adjusted *p* value = 0.633). A significant interaction was found between T1D and OW status (model 1, *p* = 0.030), suggesting that the negative effect of OW on perceived health change differs between people with and without T1D.

Analysis of physical functioning (Figure 1D) showed that T1D alone did not significantly affect scores compared to controls (model 1, *p* = 0.814). In contrast, OW had a strong negative impact, with affected participants reporting greater functional limitations than normal‐weight individuals; model 1, *p* < 0.001; adjusted *β* = −0.347 in both people with and without T1D (*p*‐value for interaction = 0.224). This significance was preserved in the fully adjusted model (model 2, adjusted *p* value = 0.035, adjusted *β* = −0.493).

OW was also associated with greater limitations in daily and work activities due to physical health (model 1, *p* = 0.035; adjusted *β* = −0.159), in both people with and without T1D (*p*‐for interaction *p* = 0.106) (Figure 1E). The association between T1D and limitations in daily and work activities did not reach significance (model 1, *p* = 0.073). This was preserved also in the fully adjusted model (model 2, *p* = 0.042, adjusted *β* = −0.210 for OW; *p* = 0.089; adjusted *β* = −0.080 for T1D).

T1D or OW were not associated with role limitations caused by emotional problems, vitality scores, or the SF‐36 mental health domain, in both model 1 and 2 (Figure 1F–H).

**FIGURE 2:**
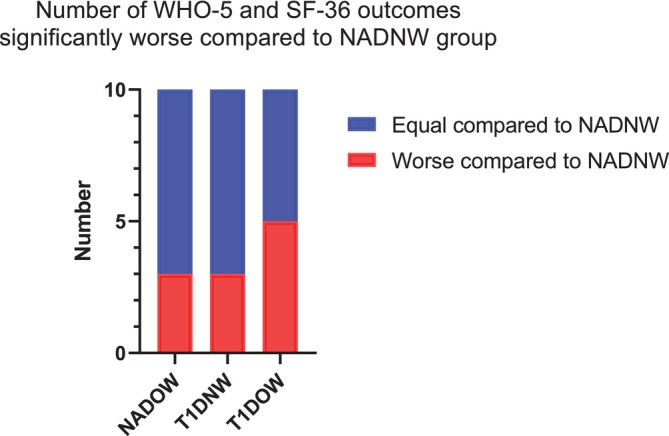
Number of WHO‐5 and SF36 outcomes worse than NADNW group. NAD, non‐diabetic; NADNW, non‐diabetic normal weight; NADOW, non‐diabetic overweight/obese; NW, normal weight; OW, overweight/obesity; SF‐36, Short Form Health Survey 36; T1D, autoimmune diabetes; T1DNW, T1D normal weight; T1DOW, T1D overweight/obese; WHO‐5, World Health Organization‐Five Well‐Being Index.

Social functioning (Figure 1I) was significantly impaired in T1D compared to controls (*p* = 0.028; adjusted *β* = −0.170), both in people with and without OW (*p*‐value for interaction = 0.918), although the significance was lost after full adjustment (model 2, adjusted *p*‐value = 0.982). Weight status showed no significant effect (model 1 *p*‐value = 0.672, model 2 *p*‐value = 0.466). Both T1D and OW were significantly associated with higher body pain (model 1 *p* = 0.043; adjusted *β* = −0.147 and *p*‐value = 0.006; adjusted *β* = −0.195, respectively), with no interaction between the two conditions (*p* = 0.933) (Figure 1L). These significances were preserved after full adjustment (model 2, for T1D = adjusted *p*‐value = 0.030 adjusted *β* = −0.488; for OW = adjusted *p*‐value = 0.008, adjusted *β* = −0.516). Overall, the presence of OW alone was associated with 3 out of 10 outcomes from WHO‐5 and SF‐36 significantly worse than the healthy control group (NADNW); similarly, the presence of T1D alone was associated with 3 out of 10 outcomes significantly worse than NADNW; noteworthy, the coexistence of OW with T1D was associated with 5 outcomes significantly worse than NADNW (Figure 2).

### 3.3 Impact of Overweight/Obesity on Distress and Empowerment in Autoimmune Diabetes

#### 3.3.1 Diabetes‐Related Distress (DDS)

**FIGURE 3:**
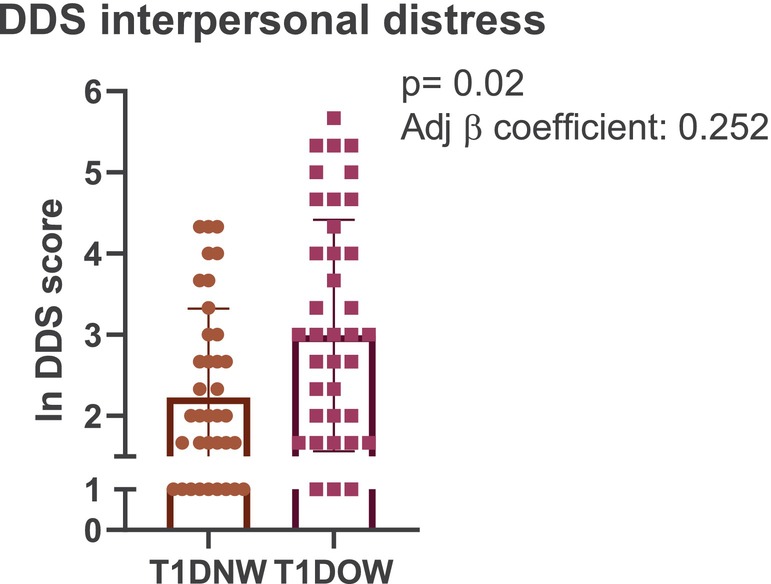
Interpersonal distress in individuals with autoimmune diabetes with normal weight (T1DNW) and overweight/obesity (T1DOW), assessed using the Diabetes Distress Scale (DDS).

OW significantly aggravated interpersonal distress (*p* = 0.020; adjusted *β* = 0.252), a PROM assessing the effect of diabetes on family and social relationships, after adjustment for confounders (Figure 3). Conversely, weight status was not significantly associated with other DDS domains: physician‐related distress (dissatisfaction with medical care and communication difficulties), regimen‐related distress (daily self‐management challenges), or emotional distress (emotional burden of diabetes). Male sex and older age were associated with greater physician‐related distress (male sex: *p* = 0.011; adjusted *β* = 0.299; age: *p* = 0.039; adjusted *β* = 0.253).

#### 3.3.2 Impact on Empowerment (DES‐SF)

DES‐SF analysis showed no significant differences in perceived empowerment between people with T1D with and without OW, after adjustment for confounders. None of the three domains—psychosocial management, goal‐setting/achievement and self‐care—were influenced by weight status. Multivariate analyses showed that males had higher scores in psychosocial management (*p* = 0.027; adjusted *β* = 0.261) and goal‐setting/achievement (*p* = 0.017; adjusted *β* = 0.287).

#### 3.3.3 Type 1 Diabetes‐Specific Distress (T1‐DDAS)

T1‐DDAS analysis revealed no significant associations between OW and the ten domains of type 1 diabetes distress, including financial worries, interpersonal challenges, management difficulties, shame, hypoglycaemia concerns, healthcare quality, lack of resources, technology challenges, burden to others and worries about complications, after adjustment by confounders. Insulin pump use was associated with greater management difficulties (*p* = 0.013; adjusted *β* = 0.215), while longer disease duration was associated with fewer management difficulties (*p* = 0.013; adjusted *β* = −0.327), consistent with adaptive processes over time. For the shame domain, male sex was associated with lower scores (*p* = 0.006; adjusted *β* = −0.219).

## 4 Discussion

This study demonstrates that T1D and OW have distinct patterns of impact on HRQoL, with T1D primarily affecting psychosocial domains and OW mainly compromising physical aspects. Moreover, the two conditions show a selective synergistic effect on body pain, while the analysis of diabetes‐specific instruments reveals an impact of OW primarily on interpersonal distress. Finally, the coexistence of both OW and T1D has an overall greater detrimental effect on quality of life in comparison to OW and T1D alone.

Understanding the combined impact of T1D and OW on QoL represents a critical gap in the scientific literature, despite the growing prevalence of ‘double diabetes’, affecting up to 55% of patients with T1D [8, 9]. Existing studies have documented the impact of diabetes and obesity on quality of life separately, but not their interaction. The evidence from the present study is consistent with the DAWN studies, which documented the pervasive impact of diabetes on psychosocial domains, with 44.6% of patients reporting significant distress [2]. Our findings confirm this pattern, showing significant impairment in emotional well‐being and social functioning, with 14.5% of patients with T1D presenting scores indicative of likely depression. With regards to OW, our findings align with the meta‐analysis by Ul‐Haq et al. [4], which showed that adults with obesity experience significant impairment in the physical aspects of QoL. Our results further show that T1D and OW are both independent risk factors for bodily pain as assessed by the SF‐36, showing that OW further worsens an already compromised pain in people with T1D. This can be explained by distinct pathophysiological mechanisms: T1D causes peripheral neuropathy through chronic hyperglycaemia and oxidative stress [19], while OW contributes to systemic inflammation through pro‐inflammatory cytokines (TNF‐α, IL‐6 and IL‐1β) released by visceral adipose tissue [20]. Our results are in line with those published by Steenhakers et al. [10] where obesity in T1D was associated with lower scores for bodily pain, physical functioning, general health and vitality evaluated with SF‐36. The selective impact of OW on interpersonal distress in people with T1D might be explained by the ‘double stigma’ theory [6]. People with T1D and OW experience stigmatisation on two fronts: the social perception of diabetes and the stigma associated with OW [7, 21]. This dual stigmatisation can amplify interpersonal tensions and impair the quality of family and social relationships. Furthermore, diabetes management may be more complex in people affected by OW, generating additional family conflicts and increasing the perceived burden on interpersonal relationships. The gender differences identified in the present study show a complex, domain‐specific pattern: while men reported greater psychosocial empowerment (DES‐SF) and less diabetes‐related shame (T1‐DDAS), they paradoxically also reported greater distress in their relationships with healthcare professionals (DDS). This profile indicates differentiated and non‐univocal coping strategies between genders, suggesting complex mechanisms that require personalised psychosocial support approaches in the management of T1D. Other findings include the paradoxical association between insulin pump use and greater perceived management difficulties, which might be explained by high expectations toward technology and the initial complexity of using the device [22, 23]. In contrast, longer disease duration was associated with fewer management difficulties, consistent with findings that show management challenges are often greatest in the early years after diagnosis, highlighting the importance of intensified support interventions [24, 25]. This study has some limitations. Since this is a cross‐sectional study, it does not allow conclusions on cause‐effect relationships between OW and deterioration in QoL. Moreover, although the study design aimed to ensure homogeneity among groups in terms of sex, age and BMI, differences in questionnaire response rates led to a small but statistically significant difference in BMI between groups. In particular, the T1DOW group showed a slightly higher BMI compared with the NADOW group (T1DOW: BMI = 29.7 [26.71–32.45] kg/m^2^ vs. NADOW: BMI = 28.2 [24.9–30.6] kg/m^2^; *p* = 0.004). Although we cannot exclude that this difference may have influenced the results, at least marginally, we also highlight that this difference, although statistically significant, is small in its absolute value and that all patients in both groups were affected by a condition of OW/OB.

Similarly, participants in the NADNW group were younger than those in the T1DNW and NADOW groups. To address this potential bias, age and sex, as well as hypertension, dyslipidaemia and anti‐diabetes therapies were included as confounding factors in the regression models.

Furthermore, as emotional well‐being and HRQoL are strongly influenced by socioeconomic factors such as income, education and deprivation, the absence of data about socioeconomic status (SES) limits the interpretation of psychosocial outcomes. Future studies should include SES indicators to better account for social determinants of health.

Finally, another limitation of the study is the use of general quality‐of‐life questionnaires, rather than instruments specifically designed for populations with overweight or obesity [26]. While we believe that the assessments performed are sufficient to capture certain aspects of the impact of overweight in autoimmune diabetes, future studies would benefit from incorporating questionnaires tailored to this vulnerable population. On the other hand, the integrated assessment strategy, combining both generic and diabetes‐specific instruments, provided a multidimensional understanding of the impact of T1D and OW on HRQoL.

Considering the growing prevalence of OW among people with T1D and the importance of QoL as a clinical outcome [27], our findings have immediate practical implications. People with T1D require systematic screening for psychosocial aspects, and those also affected by OW may further benefit from integrated approaches to pain management and psycho‐educational interventions involving family support.

## 5 Conclusions

In conclusion, this study highlights that T1D and OW determine distinct patterns of impact on QoL. OW in people with T1D selectively affects interpersonal distress, while both conditions are independent and additive risk factors for body pain. The findings support the need for integrated clinical management that considers not only metabolic control but also the multidimensional aspects of well‐being in people with T1D and OW, suggesting personalised therapeutic approaches that take gender differences into account in order to optimise HRQoL in this growing population.

## Funding

This work was supported by European Union‐Next Generation EU, Mission 4, Component 1, CUP B53D23021820001, Italian Ministry of University and Research, PRIN project #2022NS7PRM.

## Conflicts of Interest

E.M.: NovoNordisk, Eli‐Lilly, Medtronic. The other authors declare no conflicts of interest.

## Supporting information


**Figure S1:** CONSORT diagram.

## Data Availability

The data that support the findings of this study are available from the corresponding author upon reasonable request.
