# Supplementary material for: The Burden of Obesity and Type 1 Diabetes: Distinct and Synergistic Effects to Worsen Perceived Health and Psychosocial Distress
Source: Diabetes Obes Metab. 2026 Mar 9;28(5):4335–43. doi: 10.1111/dom.70629 (PMC13071217; doi:10.1111/dom.70629)
Supplement: Supplementary file 1 [file DOM-28-4335-s001.odt]

Figure S1. CONSORT Diagram. Abbreviations: WHO-5, the World Health Organization-five well-being index; SF-36, Short Form (36) Health Survey; DES-SF, Diabetes Empowerment Scale – Short Form; T1-DAS ,Type 1 Diabetes Stigma Assessment Scale; DDS, Diabetes Distress Scale; NADNW, non diabetes normal weight; NADOW, non diabetes with ovwerweight/obesity; T1DNW, type 1 diabetes with normal weight; T1DOW, type 1 diabetes with overweight/obesity.
